# The influence of pregnant women’s personality traits and well-being on internet-based decision-making and coping styles with stress

**DOI:** 10.1186/s12884-026-08878-9

**Published:** 2026-03-03

**Authors:** Nuran Nur Aypar Akbag, Yasemin Sanli

**Affiliations:** 1 Midwifery Department, Faculty of Health Sciences, Sinop University, Sinop, Turkey; 2 Department of Midwifery, Faculty of Health Sciences, Karamanoglu Mehmetbey University, Karaman, Turkey; 3 Osmaniye Neighbourhood Universite Street No:52L, 57000 Sinop, Türkiye

**Keywords:** Pregnancy, internet, coping with stress, personality traits, well-being, nursing

## Abstract

**Background:**

The worldwide utilization of computers and the internet has transformed the way in which individuals access information. This study aims to examine the associations between pregnant women’s personality traits, well-being, and their internet-based decision-making and stress coping styles.

**Methods:**

The study was designed as a descriptive correlational type. The research was conducted between October 2023 and May 2024 and included 231 pregnant women. Data were collected using a Personal Information Form, the Cervantes Personality Scale, the World Health Organization Five Well-Being Index (1998 version), the Internet Decision-Making Scale in Pregnancy, and the Stress Coping Styles Scale.

**Results:**

A negative correlation was found between the total score of the Internet Decision-Making Scale in Pregnancy and the extroversion/introversion personality trait (*r*=-0.178, *p* = 0.007), whereas a positive significant correlation was observed with the consistency/inconsistency trait (*r* = 0.180, *p* = 0.006). Statistically significant relationships were observed at different levels and in different directions among the subdimensions of the Stress Coping Styles Scale, with the exception of the “Seeking Social Support” subdimension. No significant associations were found between WHO-5 well-being and the Internet Decision-Making Scale in Pregnancy or the Stress Coping Styles Scale.

**Discussion:**

The findings suggest that internet use may be increasingly involved in the decision-making processes of pregnant women and may be associated with individual personality traits.

**Conclusion:**

Healthcare professionals are encouraged to integrate digital health literacy interventions into antenatal care and to recommend validated online information platforms to promote informed and safe internet-based decision-making among pregnant women.

## Introduction

With the widespread use of computers and the internet globally, the way people access information has undergone a significant transformation. According to the “Digital 2024” report, which presents social and digital statistics from We Are Social, there are 74.41 million active internet users in Türkiye [1]. Furthermore, data from the Turkish Statistical Institute indicate that internet usage in Türkiye has reached 88.8%, with users frequently searching for health-related information online [2–4]. Internet use has become increasingly common during pregnancy, a period marked by both uncertainty and anticipation [5–9]. Topics commonly searched include the birth process, delivery methods, fetal development, newborn care, breastfeeding, and physiological changes during pregnancy [9–12]. It is well established that individuals’ sociodemographic characteristics [13, 14], as well as personality traits [15], significantly influence internet use. Aktas and colleagues (2025) found that age, income, and pregnancy planning status significantly influenced the level of internet-based decision making [16]. These findings suggest that pregnant women’s decision-making processes may be positively or negatively affected by the information they encounter online. Digital decision support tools (such as web-based platforms, mobile applications, and interactive software) have become increasingly widespread in recent years and have been shown to enhance access to information and reduce decisional conflict. Moreover, digital decision support tools have been reported to improve the quality of prenatal decision making [17]. In addition, growing evidence indicates that internet-based decision making among pregnant women is increasing and positively affecting their quality of life [18–21].

**Fig. 1:**
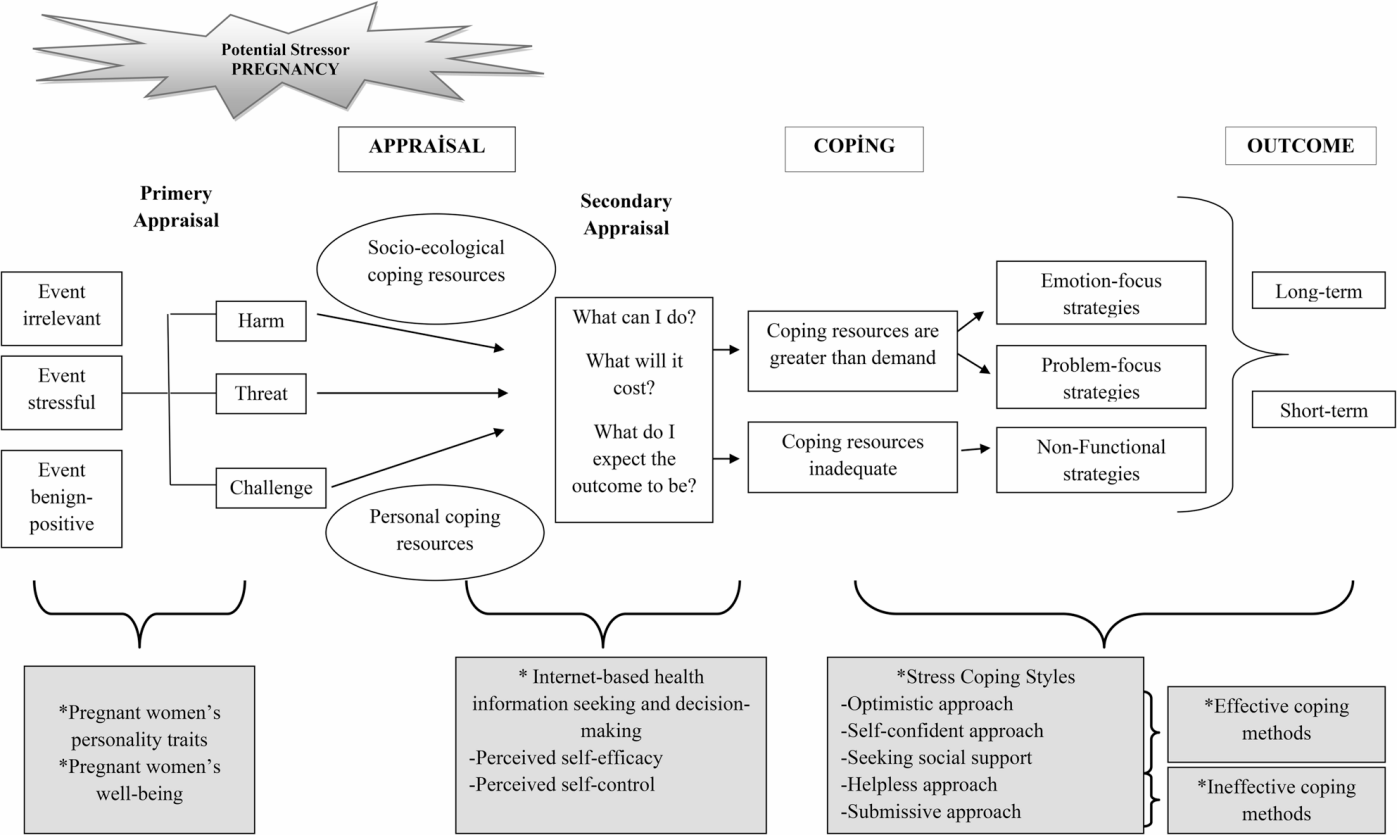
Conceptual framework of personality traits, well-being, internet-based decision-making, and stress coping during pregnancy

The concept of well-being, as defined by the World Health Organization, is not merely the absence of illness, but rather a state of physical, mental, and social well-being [22]. Well-being is considered a factor that may significantly influence individuals’ decision-making processes and coping styles. may be perceived as a threat or a challenge due to increased uncertainty and heightened information needs, thereby necessitating the use of coping strategies. In this context, the Stress and Coping Process Model proposed by Lazarus and Folkman (1984) becomes particularly relevant [23]. According to this model, stress is defined as a dynamic relationship between the individual and their environment. Cognitive appraisal and coping are central concepts within stress theory [24]. Two types of cognitive appraisal processes are described: primary appraisal and secondary appraisal. Primary appraisal refers to the individual’s evaluation of whether a situation poses a threat or challenge. When individuals do not perceive themselves to be in danger, they appraise the situation as positive or irrelevant; however, when they perceive a threat, the situation is appraised as stressful [24]. Before proceeding to the secondary appraisal process, socio-ecological and personal resources come into play. Socio-ecological resources refer to perceived support networks within the individual’s environment, including social support from family and friends. Personal coping resources include physical health, beliefs and ideology, personal values, expectations, previously experienced coping mechanisms, financial resources, and parenting skills. During secondary appraisal, individuals evaluate the resources available to them to prevent harm or manage the perceived threat [25]. In this process, individuals draw on feelings of trust or anger toward themselves and others, perceived coping capacity, and expectations for the future to develop coping strategies [25, 26] (Fig. 1). Psychological well-being in pregnancy; it is known that the mother changes the way she perceives stress factors, accelerates the physiological recovery process and strengthens the coping with stress by developing emotional regulation strategies [27]. In addition, although psychological well-being is influenced by multiple factors (such as social support, health perception, risk perception, and cultural values) it can profoundly influence decision-making mechanisms related to childbirth and postpartum care during pregnancy [28, 29].

In this context, it has become necessary to examine the extent to which pregnant women’s personality traits and well-being are associated with their internet-based decision-making and stress coping styles. Accordingly, the aim of the present study is to evaluate the relationships between pregnant women’s personality traits and well-being and their internet-based decision-making and stress coping styles.

### Research questions

- Q1: Is there a relationship between pregnant women’s personality traits and their internet-based decision-making and stress coping styles?
- Q2: Is there a relationship between pregnant women’s well-being and their internet-based decision-making and stress coping styles?
- Q3: Is there a relationship between internet-based decision-making and stress coping styles among pregnant women?

## Materials and methods

### Type of the study

This study was designed as a descriptive correlational study and conducted among pregnant women using an online data collection method.

### Study setting and sample of the study

Participants who met the inclusion criteria were selected using the purposive sampling method. The study population consisted of women reached via various social media platforms (WhatsApp, Twitter, Instagram, Facebook, Pinterest, and Snapchat) between October 2023 and May 2024. The final sample size was 231. The G* Power 3 software program was used to determine the adequacy of the sample size and the power of the study [30]. Based on the study data, with a total sample size of 231 and a correlation coefficient of *r* = 0.197 (*p* = 0.003) obtained from the correlation analysis between the total mean scores of the Internet Decision-Making Scale in Pregnancy and the Stress Coping Styles Scale, the statistical power of the study was calculated as 0.92 for the correlation analysis at a 5% significance level (α = 0.05).

Pregnant women were included if they were in their second or third trimester, whether primigravida or multigravida, carrying single or multiple fetuses, actively using the internet (i.e., owning a smartphone with internet access or using a computer with internet at home or work), and voluntarily agreed to participate. Those with high-risk pregnancies, defined as having systemic illnesses (such as obesity, hypertension, diabetes mellitus, epilepsy, renal and autoimmune disorders, coagulation disorders, anemia, smoking, or alcohol use) or conditions predisposing to preterm birth (such as premature rupture of membranes, multiple pregnancies, intrauterine growth restriction, cervical insufficiency, placental abruption, or other placental disorders) were excluded from the study.

### Data collection

Participants were recruited using a purposive sampling approach through online platforms. The study link, created using Google Forms, was disseminated by the researchers via social media channels, including WhatsApp, Twitter, Instagram, Facebook, Pinterest, and Snapchat. The survey link was shared in pregnancy-related groups, forums, and individual networks where potential participants who met the inclusion criteria were likely to be present. Participation was entirely voluntary, and no incentives were offered. The online questionnaire was accessible only to individuals who met the eligibility criteria. The first page of the survey included an electronic informed consent form detailing the purpose of the study, procedures, and inclusion and exclusion criteria. Participants were required to confirm their consent by selecting the “I agree” option before proceeding to the survey questions. No personally identifiable information, including names, contact details, or email addresses, was collected, and all responses were recorded anonymously.

### Data collection tools

The data were collected using the Personal Information Form, the Cervantes Personality Scale (CPS), the WHO (Five) Well-Being Index (1998 version), the Internet Decision-Making Scale in Pregnancy (IDMSP), and the Stress Coping Styles Scale (SCSS).

### Personal information form

This form included demographic information (age, marital status, family type, education level, economic status, etc.) and obstetric information (number of children, number of miscarriages and curettage procedures, gestational week, etc.). It also included questions assessing internet usage.

### WHO (Five) well-being index (1998 version)

Developed by the World Health Organization [31] and validated in Turkish by Eser et al. (2019), this five-item Likert-type scale assesses mood status over the past two weeks [32]. Each item is rated from 0 to 5, where 0 indicates the absence of positive feelings and 5 denotes constant positive feelings. The total score is multiplied by four to yield an index score out of 100. Scores below 50 suggest a possible depressive mood, while scores below 25 indicate a high probability of depressive mood. The percentage score is used to monitor variability in well-being, with a 10% change considered significant.

### Cervantes Personality Scale (CPS)

Originally developed by Castelo-Branco et al., the Turkish validity and reliability study of the scale was conducted by Bal and Şahin (2011) [33]. The scale comprises 20 items rated on a six-point Likert scale. Higher scores in each subdimension indicate higher levels of introversion, emotional instability (neuroticism), and inconsistency, while lower scores suggest extroversion, emotional stability, and consistency. In the present study, Cronbach’s alpha values were 0.61 for extroversion/Introversion, 0.72 for emotional stability/instability (neuroticism), and 0.74 for consistency/Inconsistency.

### Internet Decision-Making Scale in Pregnancy (IDMSP)

Developed by Koyun and Erbektaş (2018), this ten-item scale measures the influence of the internet on decision-making during pregnancy [34]. It consists of two subdimensions: “perceived self-efficacy” (items 1–5) and “perceived self-control” (items 6–10). Scores range from 10 to 50, with higher scores indicating a greater influence of the internet on decision-making. In the present study, Cronbach’s alpha values were 0.77 for perceived self-efficacy, 0.83 for perceived self-control, and 0.87 for the total scale.

### Stress Coping Styles Scale (SCSS)

Adapted into Turkish by Şahin and Durak (1995), this 30-item four-point Likert scale includes five subdimensions: optimistic approach, self-confident approach, seeking social support, helpless approach, and submissive approach [35]. Higher scores in each subdimension indicate more frequent use of that particular coping strategy. The first three subdimensions are grouped under effective coping methods, while the last two represent ineffective coping methods. Total scores are not calculated. In this study, the Cronbach’s alpha values for the subdimensions were 0.62 (optimistic approach), 0.80 (self-confident approach), 0.41 (seeking social support), 0.80 (helpless approach), and 0.72 (submissive approach).

### Data analysis

The data were analyzed using SPSS 25.0 (Statistical Package for Social Sciences for Windows). Descriptive statistics (frequency, percentage, mean, and standard deviation) were used to analyze sociodemographic and obstetric characteristics. For the scales, arithmetic mean, standard deviation, minimum, and maximum values were calculated. Skewness and kurtosis values were used to test for normality, which confirmed a normal distribution. Pearson’s correlation analysis was performed to determine the relationships between the study variables. The significance level (α) was set at 0.05, with *p* < 0.05 considered statistically significant.

### Ethical considerations

Ethical approval was obtained from the university’s human research ethics committee. Informed consent was obtained online from participants, with detailed information about the study’s purpose and inclusion/exclusion criteria. The “Confidentiality and Privacy Protection” principle was strictly followed, and all data were kept confidential. This study was conducted in accordance with the Declaration of Helsinki.

## Results

The average age of the pregnant women who participated in the study was 29.66 ± 4.92 years. It was found that 42% (*n* = 97) of the women had undergraduate or postgraduate education. The majority of participants (84%, *n* = 194) were multigravida, and 74% (*n* = 171) reported that their pregnancies were planned and desired. Regarding internet usage frequency, 27.7% (*n* = 64) stated that they used the internet ten times or more daily. When asked about their reasons for using the internet during pregnancy, the most common response (42%) was “easy access to information” (Table 1).

**Table 1:** Demographic and Obstetric Characteristics of the Pregnant Women (*n* = 231)

Characteristics	Mean	SD^†^	Min-Max
Yaş	29.66	4.92	19-43
	*n*	%	
Education level			
Primary school	29	12.6	
High school	58	25.1	
Associate degree	47	20.3	
Undergraduate and postgraduate	97	42.0	
Income status			
Income less than expenses	16	6.9	
Income equal to expenses	167	72.3	
Income more than expenses	48	20.8	
Family type			
Nuclear family	204	88.3	
Extended family	27	11.7	

	Mean	SD	Min-Max
Gestational week	32.40	6.65	6-41
	*n*	%	
Gravida			
Primigravida	37	16.0	
Multigravida	194	84.0	
History of abortion/curettage			
Yes	149	64.5	
No	82	35.5	
Whether the pregnancy was desired			
Yes	171	74.0	
No	60	26.0	
Receiving prenatal care			
Yes	103	44.6	
No	128	55.4	
Frequency of internet use			
1–3 times a day	50	21.6	
3–6 times a day	61	26.4	
6–9 times a day	56	24.2	
10 times a day or more	64	27.7	
Reason for internet use during pregnancy			
Ease of accessing information	97	42.0	
Finding answers to all questions	38	16.5	
Access to information at any time	96	41.6	

^†^*SD* Standard deviation

The average score for the Well-Being Index among participants was 58.68 ± 19.70. The subscale scores for the Cervantes Personality Scale were as follows: Extroversion/Introversion: 13.11 ± 5.38, Emotional Stability/Instability (Neuroticism): 17.78 ± 6.14, and Consistency/Inconsistency: -18.18 ± 5.67. The average total score for the Internet Decision-Making Scale in Pregnancy (IDMSP) was 33.52 ± 6.77. Regarding stress coping styles, the average scores for effective coping methods were: Optimistic Approach: 14.06 ± 2.58, Self-Confident Approach: 21.10 ± 3.57, and Seeking Social Support: 10.94 ± 1.77. For ineffective coping methods, the averages were: Helpless Approach: 17.22 ± 4.17 and Submissive Approach: 12.26 ± 3.36 (Table 2).

**Table 2:** Descriptive Statistics of Scales (*n* = 231)

	*n*	%
WHO (Five) Well-Being Index Classification		
Normal mood (51–100 points)	154	66.7
Depressive mood (26–50 points)	63	27.3
High likelihood of depressive mood (0–25 points)	14	6.1

	Mean±SD^†^	Min-Max
WHO (Five) Well-Being Index	58.68±19.70	8.00-100.00
Cervantes Personality Scale		
Extroversion/Introversion	13.11±5.38	0.00-26.00
Emotional stability/instability (neuroticism)	17.78±6.14	0.00-35.00
Consistency/Inconsistency	-18.18±5.67	-30.00- 00.00
Internet Decision-Making Scale in Pregnancy		
Perceived self-efficacy	16.29±3.62	7.00-25.00
Perceived self-control	17.24±3.88	7.00-25.00
Total	33.52±6.77	16.00-50.00
Stress Coping Styles Scale		
Effective coping methods		
Optimistic approach	14.06±2.58	6.00-20.00
Self-confident approach	21.10±3.57	12.00-28.00
Seeking social support	10.94±1.77	7.00-15.00
Ineffective coping methods		
Helpless approach	17.22±4.17	8.00-31.00
Submissive approach	12.26±3.36	6.00-22.00

^†^*SD* Standard deviation

Table 3 presents the relationship between CPS scores and IDMSP and SCSS scores. A weak positive correlation was identified between perceived self-efficacy (IDMSP) and the Consistency/Inconsistency personality trait (*r* = 0.229, *p* = 0.000). A weak negative correlation was observed between perceived self-control and Extroversion/Introversion (*r*=-0.215, *p* = 0.001). The total IDMSP score was negatively correlated with Extroversion/Introversion (*r*=-0.178, *p* = 0.007) and positively correlated with Consistency/Inconsistency (*r* = 0.180, *p* = 0.006). Among effective coping styles, the Optimistic Approach and Self-Confident Approach were found to be significantly associated with personality traits. However, Seeking Social Support was not significantly correlated with personality traits. Regarding ineffective coping styles, significant correlations were observed between Emotional Stability/Instability and Consistency/Inconsistency (Table 3).

**Table 3:** Correlations Between Internet Decision-Making, Stress Coping Scores and Personality Traits (*n* = 231)

Cervantes Personality Scale						
	Extroversion/Introversion		Emotional stability/instability (neuroticism)		Consistency/Inconsistency	
Internet Decision-Making Scale in Pregnancy	r^†^	*p*	*r*	*p*	*r*	*p*
Perceived self-efficacy	-0.102	0.121	-0.012	0.856	0.229	0.000*
Perceived self-control	-0.215	0.001*	-0.007	0.910	0.101	0.127
Total	-0.178	0.007*	-0.002	0.974	0.180	0.006*
Stress Coping Styles Scale						
Optimistic approach	-0.236	0.000*	-0.089	0.177	-0.130	0.048*
Self-confident approach	-0.197	0.003*	-0.145	0.027*	-0.194	0.003*
Seeking social support	-0.045	0.498	-0.062	0.351	-0.035	0.598
Helpless approach	0.097	0.140	0.359	0.000*	0.254	0.000*
Submissive approach	0.110	0.095	0.268	0.000*	0.220	0.001*

† *r* = Pearson correlation coefficient, *p< 0.05

Well-being scores measured by the WHO (Five) Well-Being Index were not significantly associated with the total or subscale scores of IDMSP and SCSS (Table 4).

**Table 4:** Correlations Between Internet Decision-Making, Stress Coping Scores and Well-Being (*n* = 231)

	WHO (Five) Well-Being Index	
	*r* ^†^	*p*
Internet Decision-Making Scale in Pregnancy		
Perceived self-efficacy	0.089	0.177
Perceived self-control	-0.011	0.867
Total	0.041	0.532
Stress Coping Styles Scale		
Optimistic approach	0.092	0.164
Self-confident approach	-0.041	0.531
Seeking social support	0.039	0.557
Helpless approach	0.093	0.157
Submissive approach	0.047	0.474

† *r* = Pearson correlation coefficient, *p< 0.05

A weak positive correlation was found between perceived self-efficacy (IDMSP) and the Helpless Approach (*r* = 0.180, *p* = 0.006). Perceived self-control was positively correlated with Optimistic Approach (*r* = 0.175, *p* = 0.008) and Self-Confident Approach (*r* = 0.176, *p* = 0.007) among effective coping styles, and also with Helpless Approach (*r* = 0.176, *p* = 0.007) among ineffective coping styles. A statistically significant correlation was found only between the total IDMSP score and the Helpless Approach (*r* = 0.197, *p* = 0.003) (Table 5).

**Table 5:** Correlations Between Internet Decision-Making and Stress Coping Styles (*n* = 231)

	Stress Coping Styles Scale									
	Effective Coping Methods						Ineffective Coping Methods			
	Optimistic approach		Self-confident approach		Seeking social support		Helpless approach		Submissive approach	
	*r* ^†^	*p*	*r*	*p*	*r*	*p*	*r*	*p*	*r*	*p*
Internet Decision-Making Scale in Pregnancy										
Perceived self-efficacy	-0.013	0.846	0.022	0.743	0.048	0.472	0.180	0.006*	0.055	0.402
Perceived self-control	0.175	0.008*	0.176	0.007*	0.044	0.501	0.176	0.007*	0.114	0.083
Total	0.093	0.157	0.112	0.088	0.051	0.441	0.197	0.003*	0.095	0.149

†*r*: Pearson correlation coefficient, *p < 0,05

## Discussion

As in many parts of the world, internet and social media usage is steadily increasing in Türkiye, often surpassing global averages [36]. Health-related searches are driven by factors such as privacy, convenience, and instant access, and women are known to use the internet more frequently than men. During pregnancy a time when the need for information is heightened frequent use of the internet and social media may significantly influence pregnant women’s health-related decision-making processes [37]. Health literacy is an important competency for distinguishing the reliability of information obtained from the internet [38]. Findings indicate that limited health literacy among pregnant women is associated with increased unhealthy behaviors and decreased adherence to drug treatments [39, 40]. However, while increased internet use is thought to enhance pregnant women’s health literacy, decision-making through the internet has been reported to decrease [41]. While these findings reflect more effective evaluation of information reliability at higher levels of health literacy, they may be explained by decreased reliance on the internet as an authority in decision-making.

In this study, pregnant women who decision-making via internet were found to be more extroverted yet more inconsistent. Furthermore, individuals with higher self-control in decision-making tended to exhibit more extroverted personality traits. Notably, increased self-efficacy in decision-making was associated with greater personality inconsistency. When the relationship between personality traits and decision-making styles was examined, it was found that the extroverted personality trait was more strongly associated with dependent and spontaneous decision-making styles. This can be explained by their openness to social interaction and their tendency to consult others’ opinions or make quick decisions [42]. In addition, it has been stated that high levels of neuroticism can make the process of logical decision-making challenging [42, 43]. Consistent with these findings, various studies have linked personality traits to pregnancy-related symptoms decision-making processes, depression, substance use (cigarette and alcol) [44–47]. Although the personality assessment tool used in the present study differs from those employed in earlier researches, the results support the notion that increased social engagement may influence information-seeking behaviors and enhance self-control in decision-making. Nevertheless, it should be acknowledged that the quality and reliability of health-related information obtained via the internet may be influenced by several factors, including educational level, socioeconomic status, health literacy, and cultural differences.

Pregnant women with extroverted, emotionally stable, and consistent personalities were found to associate stress coping methods such as optimism and self-confidence more frequently. In contrast, ineffective coping methods helplessness and submissiveness were more common among emotionally unstable and inconsistent individuals. Coping ability during stressful situations is particularly influenced by personality and emotional state [24, 25]. Women with personality disorders, emotional instability, or immature psychological characteristics often face greater difficulties during pregnancy. In addition, it was indicated that pregnant women with negative personality traits and poor coping mechanisms experienced more severe fear of childbirth [48]. These findings suggest that personality traits are indeed linked to stress coping styles. Extroverted, emotionally balanced, and consistent women appear to use effective coping strategies more successfully. However, in a study evaluating how pregnant women cope with stress and the factors influencing these processes, it was found that the scores for the “Self-Confident Approach” and the “Seeking Help Approach” were both high and closely aligned [49]. This finding may indicate that coping strategies in pregnancy cannot be clearly distinguished by rigid boundaries.

In the current study, no significant effect of well-being on internet-based decision-making or stress coping styles was found. According to the theory proposed by Lazarus and Folkman, well-being functions both as a resource that influences how stress is perceived during the appraisal phase and as an outcome reflecting the effectiveness of the coping process [23]. Accordingly, psychological well-being may emerge as a result of these processes rather than as a direct determinant of internet-based decision-making and coping styles during pregnancy. Psychological well-being in the healthy pregnant women included in this study may have emerged as a natural part of adaptation to the pregnancy process rather than as a direct determinant of internet-based decision-making or stress coping styles. The normal course of physiological changes during pregnancy, a low perception of medical risk, and the continuation of routine prenatal care may have helped to balance stress levels. In addition, internet-based decision-making and coping styles in healthy pregnancies may be more closely related to factors, such as individual characteristics, previous experiences, social support, digital health literacy, and pregnancy planning status.

As perceived self-control in internet-based decision-making increases, so do effective coping methods such as optimism and self-confidence. Contrary to expectations, this study found that increases in self-efficacy and self-control in internet-based decision-making were also associated with greater use of the ineffective coping method of helplessness. The internet has become a vital tool for information seeking, particularly during pregnancy, making the selection of accurate and reliable sources increasingly important [50]. The results of the current study suggest that women with ineffective coping strategies may rely on the internet both as a decision-making tool and as a means of gaining a sense of control. As pregnancy is a period of profound physiological and psychological change, it may represent a developmental crisis or critical period; therefore, identifying pregnant women’s coping strategies is essential for providing appropriate care [51]. However, the suitability and safety of internet-based practices for each individual remain open to debate. In particular, inaccurate online information may lead to poor decision-making and increased stress, thereby intensifying feelings of helplessness. Healthcare professionals should inform pregnant women about safe internet use and guide them toward reliable online resources. In addition, initiatives and training programs aimed at improving digital health literacy should be developed to enable pregnant women to critically evaluate online health information.

### Strengths and limitations

The primary strength of this study lies in its originality, as there are limited studies examining the combined relation of personality traits and well-being on internet-based decision-making and stress coping styles in pregnant women. By integrating these variables within a theoretically grounded framework, the study offers a novel perspective and contributes to a more comprehensive understanding of coping and decision-making processes during pregnancy.

Several limitations should be acknowledged. First, purposive sampling and online data collection via social media may have introduced selection bias, potentially overrepresenting pregnant women who are more educated, internet-active, or digitally literate. Therefore, the findings may not be generalizable to all pregnant women. Second, data were collected within a specific time frame, which may limit temporal generalizability. Third, although the study is theoretically grounded, potential confounders such as social support and perceived health could not be statistically controlled, constituting a methodological limitation.

Finally, the Cronbach’s alpha coefficient of the “Seeking Social Support” subscale was below the acceptable threshold. This may be related to differences between the original validation sample and the current study population. Accordingly, results related to this subscale should be interpreted with caution. Future studies are recommended to use probability sampling methods, multivariate or longitudinal designs, and culturally appropriate measurement tools to enhance validity and generalizability.

## Conclusion

Based on the findings, internet-based decision-making and stress coping styles among pregnant women appear to be associated with personality traits. In contrast, well-being was not significantly associated with internet-based decision-making or stress coping styles. These results suggest that personality traits may represent one of several factors related to decision-making and coping strategies during pregnancy, without implying causal relationships. It should also be noted that well-being is a multidimensional construct that may be influenced by various confounding factors, such as perceived health status and perceived social support. Therefore, well-being cannot be directly linked to decision-making and coping processes based on the present findings alone.

In prenatal follow-up, it may be assessment pregnant women’s personality characteristics, well-being, digital health literate, health perceptions, and social support. To promote the positive and safe use of the internet, initiatives aimed at improving digital health literacy are recommended. In this context, nurses and midwives providing perinatal care should guide pregnant women toward reliable, evidence-based online health information sources. Future studies are encouraged to investigate additional factors associated with internet-based decision-making and stress coping styles during pregnancy using longitudinal or multivariate research designs.

## Data Availability

The datasets used and/or analyzed during the current study are available from the corresponding author on reasonable request.
